# A Case of Hyperparathyroidism due to a Large Intrathyroid Parathyroid Adenoma with Recurrent Episodes of Acute Pancreatitis

**DOI:** 10.1155/2017/5376741

**Published:** 2017-07-26

**Authors:** Kazunori Kageyama, Noriko Ishigame, Aya Sugiyama, Akiko Igawa, Takashi Nishi, Satoko Morohashi, Hiroshi Kijima, Makoto Daimon

**Affiliations:** ^1^Department of Endocrinology and Metabolism, Hirosaki University Graduate School of Medicine, 5 Zaifu-cho, Hirosaki, Aomori 036-8562, Japan; ^2^Department of Endocrinology and Metabolism, Odate Municipal General Hospital, 3-1 Yutaka-cho, Odate 017-8550, Japan; ^3^Department of Gastroenterological Surgery, Hirosaki University Graduate School of Medicine, 5 Zaifu-cho, Hirosaki, Aomori 036-8562, Japan; ^4^Department of Pathology and Bioscience, Hirosaki University Graduate School of Medicine, 5 Zaifu-cho, Hirosaki, Aomori 036-8562, Japan

## Abstract

We report a case of a 66-year-old woman who developed hyperparathyroidism due to a large intrathyroid parathyroid adenoma with episodes of acute pancreatitis. She had previously been treated for acute pancreatitis twice. Serum calcium was 12.4 mg/dL, and intact parathyroid hormone was 253 pg/dL. Ultrasonography and computed tomography of the neck with contrast enhancement revealed a soft tissue mass (28 mm transverse diameter) within the left lobe of the thyroid. ^99m^Tc-MIBI scintigraphy demonstrated focal accumulation due to increased radiotracer uptake in the left thyroid lobe. Left hemithyroidectomy was performed. Histopathology showed no signs of invasion, and this is consistent with parathyroid adenoma. Immunostaining was positive for expression of chromogranin A and parathyroid hormone. The patient had no episode of pancreatitis after the operation. In a patient with recurrent episodes of pancreatitis, the possibility of complication with hyperparathyroidism should be considered.

## 1. Introduction

Primary hyperparathyroidism (PHPT) is a common endocrine disorder characterized by hypercalcemia and excessive secretion of parathyroid hormone (PTH) [1]. PHPT is most commonly caused by a single adenoma of the parathyroid gland. Patients with PHPT tend to develop complications such as reduction of bone mineral density, nephrolithiasis, and gastric ulcer, which may impair quality of life [1, 2]. In the management of PHPT, parathyroidectomy of the abnormal gland is the gold standard for effective treatment. Generally, most parathyroid adenomas remain relatively small, measuring under a few centimeters and weighing less than 1 g [3]. Large or giant parathyroid adenomas are seldom seen in patients with PHPT [4], and in such cases differential diagnosis is necessary to rule out malignancy.

Acute pancreatitis may be induced by cholelithiasis and alcohol abuse in adults; however, the incidence of pancreatitis in patients with hyperparathyroidism was reported to be only 1.5% [5]. Here, we report a case of hyperparathyroidism due to a large intrathyroid parathyroid adenoma with episodes of acute pancreatitis. She had been treated for acute pancreatitis twice. However, there was no episode of pancreatitis after the operation.

## 2. Case Report

A 66-year-old woman was consulted for evaluation of hypercalcemia. She had been treated for acute pancreatitis twice (3 years and 6 months earlier) and had a long history of hypercalcemia (calcium 12.3 mg/dL (albumin 3.5 g/dL) and calcium 12.7 mg/dL (albumin 4.7 g/dL), resp.). Abdominal computed tomography (CT) had shown the presence of multiple renal stones, but not gall stones or pancreatic calcifications (not shown). She usually consumed 350 mL of beer 4 times/week. She had no palpable mass in her neck. Endocrine evaluation was performed according to relevant clinical guidelines, and the patient gave written informed consent for all tests performed. Renal function was within normal. As shown in Table 1, serum calcium was 12.4 mg/dL (reference range: 8.3–10.3), albumin 4.0 g/dL, and intact PTH (iPTH) level 253 pg/dL (reference range: 8.7–79.5). Urinary calcium/creatinine ratio was 0.39. Serum phosphorus was 2.4 mg/dL (reference range: 2.4–4.7).

**Figure 1:**
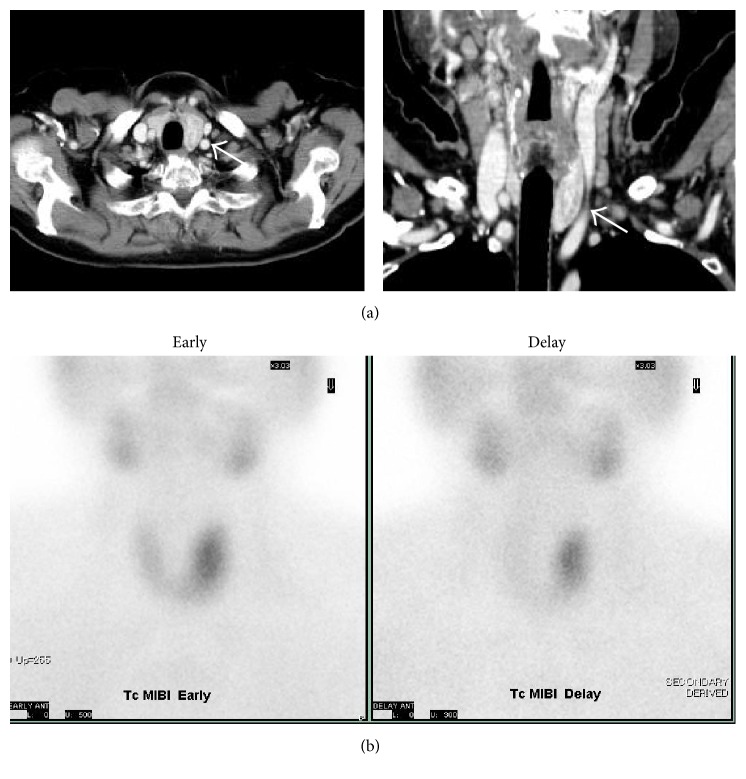
(a) Computed tomography of the neck. The scan with contrast enhancement shows a large heterogeneous soft tissue mass (28 mm transverse diameter), clearly defined, within the left thyroid lobe (white arrow). (b) ^99m^Tc-MIBI scintigraphy. Early and delayed scintigrams reveal focal accumulation of increased radiotracer uptake in the left lobe of the thyroid.

Contrast-enhanced CT of the neck revealed a heterogeneous soft tissue mass (28 mm transverse diameter), clearly defined, within the left thyroid (Figure 1(a)). T-scores of femoral and lumbar bone mineral density were −1.3 and −2.7, respectively. Technetium-99m-methoxyisobutylisonitrile (^99m^Tc-MIBI) scintigraphy demonstrated focal accumulation of increased radiotracer uptake in the left lobe of the thyroid on both early and delayed images (Figure 1(b)).

**Figure 2:**
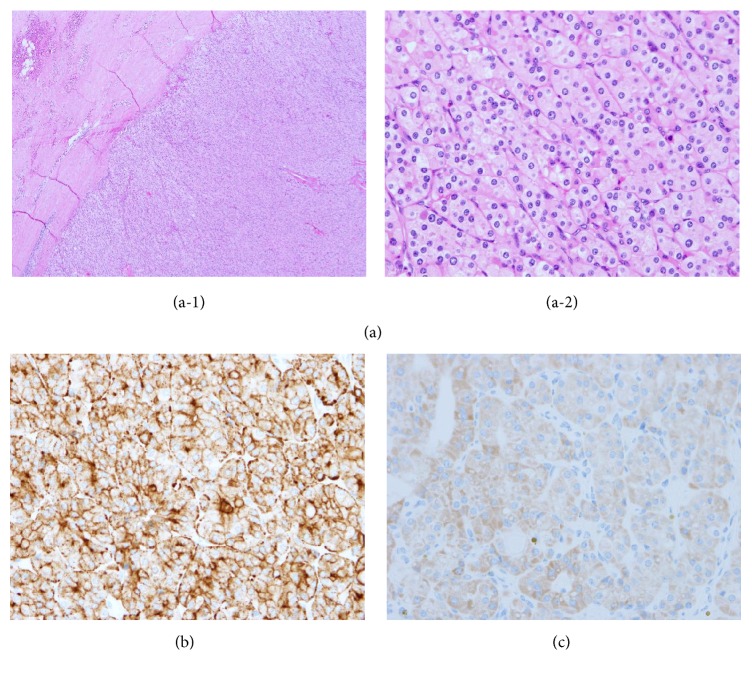
(a) Hematoxylin-eosin stained sections of the adenoma (original magnification ×4 (a-1) and ×40 (a-2)). The adenoma was composed mainly of chief cells and oxyphil cells, covered with a fibrous capsule. No signs of malignancy, such as presence of capsular invasion, angioinvasion, and invasion of the surrounding structures, were observed. (b) Immunostaining for chromogranin A (brown precipitates). Section shows expression of chromogranin A. (c) Immunostaining for PTH (brown precipitates). Section shows expression of PTH.

Left hemithyroidectomy was performed due to the clearly defined soft tissue mass within the left thyroid. Histopathology showed no signs of invasion, and this is consistent with parathyroid adenoma. The adenoma was composed mainly of chief cells and oxyphil cells, covered with a fibrous capsule (Figure 2(a)). Evaluation of chromogranin A expression showed positive chromogranin A immunostaining (Figure 2(b)). Evaluation for PTH expression showed positive PTH immunostaining (Figure 2(c)). Soon after surgery, the elevated calcium and iPTH were normalized. The patient has had no episodes of pancreatitis for one year after the operation.

## 3. Discussion

This is an unusual case of hyperparathyroidism due to a large parathyroid adenoma. This present patient had been treated for acute pancreatitis twice. Pooled clinical data suggest an association between PHPT and pancreatitis [6, 7]. Serum calcium levels in PHPT with pancreatitis were found to be higher than those in PHPT without pancreatitis [6, 8]. Acute pancreatitis may be caused by calcium-induced activation of intrapancreatic trypsinogen to trypsin. However, only a minority of patients with PHPT would develop pancreatitis. Felderbauer et al. found that mutations in the serine protease inhibitor Kazal type I (SPINK1) and cystic fibrosis transmembrane conductance regulator (CFTR) genes increase the risk for pancreatitis, and mutations in the Chymotrypsin C gene (CTRC) modulate susceptibility for pancreatitis [9, 10]. Therefore, markedly elevated serum calcium may contribute to pancreatitis, together with additional genetic or environmental insults [6].

Parathyroid adenomas usually measure less than 2 cm and weigh less than 1 g. In parathyroid lesions larger than 2 cm, the differential diagnosis between giant parathyroid adenomas and parathyroid carcinomas would be considered [11]. Parathyroid cysts or cystic adenomas often show large parathyroid ones [12]. No signs of malignancy, such as presence of capsular invasion, angioinvasion, and invasion of the surrounding structures, were observed by morphological analysis in our case. The weight or size of the adenoma may have been correlated with the functional status of the gland and the severity of biochemical abnormalities. For example, larger adenomas may be associated with a more severe form of primary hyperparathyroidism [13]. Conversely, in some cases of giant adenoma, there was no correlation with clinical symptoms or functional status [14].

The incidence of intrathyroid parathyroid adenoma is rare: true one is 0.7%, and partial one is 1.9% [15]. Imaging may miss the pathologic gland [16]. Generally, different imaging techniques, such as high resolution ultrasonography, CT, arteriography, venous sampling, and magnetic resonance imaging, have been used for detection of the abnormal parathyroid glands [17, 18]. Radionuclide imaging has also been used in the detection and localization of parathyroid adenomas. ^99m^Tc-MIBI has been used for preoperative evaluation of PHPT [19], as demonstrated in our case.

Hypercalcemia may mediate the development of pancreatitis and our patient had earlier been treated for acute pancreatitis twice. However, during short-term follow-up, she had not experienced any episodes of pancreatitis after surgery. In a patient with recurrent episodes of pancreatitis, the possibility of complication with hyperparathyroidism should be considered.

In summary, we report an unusual case of hyperparathyroidism due to a large intrathyroid parathyroid adenoma with episodes of acute pancreatitis.

## Figures and Tables

**Table 1:** General laboratory data.

	Before operation	After operation	(normal values)
Peripheral blood			
White blood cells (/*μ*L)	5690	6210	(3500–8500)
Red blood cells (/*μ*L)	3.50	3.84	(3.80–4.80 × 10^6^)
Hemoglobin (g/dL)	10.7	10.9	(11.5–15.0)
Hematocrit (%)	31.5	32.7	(34.0–45.0)
Platelets (/*μ*L)	18.1	20.5	(13.0–35.0 × 10^4^)
Blood biochemistry			
Total protein (g/dL)	6.9	7.0	(6.7–8.3)
Albumin (g/dL)	4.0	4.0	(3.9–4.9)
Total bilirubin (mg/dL)	0.8	0.6	(0.2–1.1)
Aspartate aminotransferase (U/L)	28	25	(10–35)
Alanine aminotransferase (U/L)	20	16	(7–38)
*γ*-Glutamyltranspeptidase (U/L)	28	35	(0–65)
Alkaline phosphatase (IU/L)	263	213	(104–340)
Urea nitrogen (mg/dL)	17	14	(8–25)
Creatinine (mg/dL)	0.81	0.89	(0.40–1.10)
Sodium (mmol/L)	145	143	(137–146)
Chloride (mmol/L)	112	107	(99–110)
Potassium (mmol/L)	4.4	4.0	(3.5–4.9)
Calcium (mg/dL)	12.4	9.6	(8.3–10.3)
Phosphorus (mg/dl)	2.4	3.3	(2.4–4.7)
Total cholesterol (mg/dl)	215	182	(115–220)
Triglyceride (mg/dL)	185	215	(20–150)
Plasma glucose (mg/dL)	98	132	(70–110)
Hemoglobin A1c (%)	5.1	5.8	(4.6–6.2)
Intact PTH	253.0	59.3	(8.7–79.5)

## References

[1] Bilezikian J. P., Potts J. T., El-Hajj Fuleihan G. (2002). Summary statement from a workshop on asymptomatic primary hyperparathyroidism: a perspective for the 21st century. *The Journal of Clinical Endocrinology and Metabolism*.

[2] Bringhurst F. R., Demay M. B., Kronenberg H. M., Wilsonn J. D., Foster D. W., Kronenberg H. M., Larsen P. R. (1998). Hormones and disorders of mineral metabolism. *Williams Textbook of Endocrinology*.

[3] Neagoe R. M., Sala D. T., Borda A., Mogoanta C. A., Muhlfay G. (2014). Clinicopathologic and therapeutic aspects of giant parathyroid adenomas - three case reports and short review of the literature. *Romanian Journal of Morphology and Embryology*.

[4] Krishnamurthy A., Raghunandan G., Ramshankar V. (2016). A rare case of giant parathyroid adenoma presenting with recurrent episodes of pancreatitis. *Indian Journal of Nuclear Medicine*.

[5] Bess M. A., Edis A. J., van Heerden J. A. (1980). Hyperparathyroidism and pancreatitis. Chance or a causal association?. *The Journal of the American Medical Association*.

[6] Bai H. X., Giefer M., Patel M., Orabi A. I., Husain S. Z. (2012). The association of primary hyperparathyroidism with pancreatitis. *Journal of Clinical Gastroenterology*.

[7] Bhadada S. K., Udawat H. P., Bhansali A., Rana S. S., Sinha S. K., Bhasin D. K. (2008). Chronic pancreatitis in primary hyperparathyroidism: comparison with alcoholic and idiopathic chronic pancreatitis. *Journal of Gastroenterology and Hepatology*.

[8] Shah V. N., Bhadada S. K., Bhansali A. (2014). Effect of gender, biochemical parameters and parathyroid surgery on gastrointestinal manifestations of symptomatic primary hyperparathyroidism. *Indian Journal of Medical Research*.

[9] Felderbauer P., Karakas E., Fendrich V. (2008). Pancreatitis risk in primary hyperparathyroidism: relation to mutations in the *SPINK1* trypsin inhibitor (N34S) and the cystic fibrosis gene. *The American Journal of Gastroenterology*.

[10] Felderbauer P., Karakas E., Fendrich V., Lebert R., Bartsch D. K., Bulut K. (2011). Multifactorial genesis of pancreatitis in primary hyperparathyroidism: Evidence for protective (PRSS2) and destructive (CTRC) genetic factors. *Experimental and Clinical Endocrinology and Diabetes*.

[11] Araujo Castro M., López A. A., Fragueiro L. M., García N. P. (2017). Giant parathyroid adenoma: differential aspects compared to parathyroid carcinoma. *Endocrinology, Diabetes & Metabolism Case Reports*.

[12] Ahmad M., Almohaya M., Al Johani N., Almalki M. (2017). Intrathyroidal Parathyroid Cyst: An Unusual Neck Mass. *Clinical Medicine Insights: Endocrinology and Diabetes*.

[13] Zamboni W. A., Folse R. (1986). Adenoma weight: A predictor of transient hypocalcemia after parathyroidectomy. *The American Journal of Surgery*.

[14] Power C., Kavanagh D., Hill A. D. K., O'Higgins N., McDermott E. (2005). Unusual presentation of a giant parathyroid adenoma: Report of a case. *Surgery Today*.

[15] Goodman A., Politz D., Lopez J., Norman J. (2011). Intrathyroid parathyroid adenoma: Incidence and location - The case against thyroid lobectomy. *Otolaryngology - Head and Neck Surgery*.

[16] Bahar G., Feinmesser R., Joshua B.-Z. (2006). Hyperfunctioning intrathyroid parathyroid gland: A potential cause of failure in parathyroidectomy. *Surgery*.

[17] Krubsack A. J., Wilson S. D., Lawson T. L. (1989). Prospective comparison of radionuclide, computed tomographic, sonographic, and magnetic resonance localization of parathyroid tumors. *Surgery*.

[18] Bhansali A., Masoodi S. R., Bhadada S., Mittal B. R., Behra A., Singh P. (2006). Ultrasonography in detection of single and multiple abnormal parathyroid glands in primary hyperparathyroidism: Comparison with radionuclide scintigraphy and surgery. *Clinical Endocrinology*.

[19] O'Doherty M. J., Kettle A. G., Wells P., Collins R. E. C., Coakley A. J. (1992). Parathyroid imaging with technetium-99m-sestamibi: Preoperative localization and tissue uptake studies. *Journal of Nuclear Medicine*.

